# Interaction analysis of subgroup effects in randomized trials: the essential methodological points

**DOI:** 10.1038/s41598-024-62896-1

**Published:** 2024-06-01

**Authors:** Abraham Fingerhut, Selman Uranues, Chadly Dziri, Junjun Ma, Dewi Vernerey, Hayato Kurihara, Philip Stiegler

**Affiliations:** 1 Department of General Surgery, Ruijin Hospital, Shanghai Jiao Tong University School of Medicine, Shanghai Minimally Invasive Surgery Center, Shanghai, People’s Republic of China; 2 Section for Surgical Research, Department of Surgery, Medical University of Graz, Graz, Austria; 3 Medical School of Tunis, Tunis University El Manar, Tunis, Tunisia; 4 Honoris Medical Simulation Center, Tunis, Tunisia; 5 Methodology and Quality of Life Unit, INSERM Unit. 1098, University of Besancon, Besancon, France; 6 Emergency Surgery Unit, IRCCS – Ca’ Granda - Policlinico Hospital, Via Francesco Sforza, 20122 Milan, Italy

**Keywords:** Secondary analysis, Interaction analysis, Subgroup analysis, Relative excess risk due to interaction, Attributable proportion, Randomized trial, Distal pancreatectomy, Post-operative pancreatic fistula, Hemopatch®, Health care, Medical research, Risk factors, Signs and symptoms

## Abstract

Subgroup analysis aims to identify subgroups (usually defined by baseline/demographic characteristics), who would (or not) benefit from an intervention under specific conditions. Often performed post hoc (not pre-specified in the protocol), subgroup analyses are prone to elevated type I error due to multiple testing, inadequate power, and inappropriate statistical interpretation. Aside from the well-known Bonferroni correction, subgroup treatment interaction tests can provide useful information to support the hypothesis. Using data from a previously published randomized trial where a *p* value of 0.015 was found for the comparison between standard and Hemopatch® groups in (the subgroup of) 135 patients who had hand-sewn pancreatic stump closure we first sought to determine whether there was interaction between the number and proportion of the dependent event of interest (POPF) among the subgroup population (patients with hand-sewn stump closure and use of Hemopatch®), Next, we calculated the relative excess risk due to interaction (RERI) and the “attributable proportion” (AP). The p value of the interaction was *p* = 0.034, the RERI was − 0.77 (*p* = 0.0204) (the probability of POPF was 0.77 because of the interaction), the RERI was 13% (patients are 13% less likely to sustain POPF because of the interaction), and the AP was − 0.616 (61.6% of patients who did not develop POPF did so because of the interaction). Although no causality can be implied, Hemopatch® may potentially decrease the POPF after distal pancreatectomy when the stump is closed hand-sewn. The hypothesis generated by our subgroup analysis requires confirmation by a specific, randomized trial, including only patients undergoing hand-sewn closure of the pancreatic stump after distal pancreatectomy.

Trial registration: INS-621000-0760.

## Introduction

Subgroup analysis is often observed in the medical literature^1,2^ but most surgeons are notoriously unaware of what it means or how to use it. Subgroup analysis is often done without second thought, because data dredging found a “statistically significant” value for one of the multiple possible combinations of comparisons. The rationale behind subgroup analysis is to determine how to use a particular treatment most effectively by identifying subgroups of patients who would (or would not) benefit from that treatment in specific categories, usually defined by baseline/demographic factors^3^. All too often, however, authors conducting a randomized clinical trial are tempted by post hoc analyses with the idea that results might lead to a new hypothesis and make the results of an otherwise “negative” trial valuable. Such analyses, usually generated retrospectively based on trial data, are not reliable before they can be replicated by other studies^4^.

Notwithstanding, such post hoc observations should not be automatically considered invalid or be neglected. Indeed, the rationale behind post hoc subgroup analysis is of importance: a properly selected, methodologically sound subgroup analysis can be a valid hypothesis testing ground if based on previous empirical evidence and current scientific theory.

In a recent clinical randomized trial^5^, we evaluated the efficacy, safety and tolerance of Hemopatch® added to the pancreatic stump after distal pancreatectomy (DP) in preventing clinically relevant (grades B/C according to the ISGPS classification^6,7^) postoperative pancreatic fistula (POPF). Although the difference in efficacy of the primary endpoint (POPF) was not statistically significant (24.5% vs. 16.3%, in the two groups, respectively (*p* = 0.120), the p value for the comparison between standard and Hemopatch® groups in the 135 patients who had hand-sewn pancreatic stump closure was 0.014 (17/65 and 7/70 (26.2% vs.10.0%) respectively).

However, this subgroup analysis was not pre-specified in the protocol. Subgroup analyses without pre-specification are known to be prone to statistical and methodological issues such as inflation of the type I error due to multiple testing, inadequate power, and inappropriate statistical interpretation. One of the most well-known methods to deal with this problem is the Bonferroni correction which tests each individual hypothesis at a significance level of alpha/n where alpha is the desired overall alpha level and n is the number of hypotheses^8,9^. Several guidelines and methods for the purpose, evaluation, analysis, and recording of subgroup analyses have been proposed^8–16^. Generally speaking, they feature the same recommendations: the quantity of subgroups analyzed should be small, subgroups of interest should be established on a solid biological reasoning or based on previously observed subgroup effects and proper adjustment for multiple testing should be proposed. Importantly, subgroup treatment interaction tests should be preferred to subgroup-specific tests, and all subgroups tested should be reported including whether they are preplanned or post-hoc. As all these conditions were satisfied in the previous study^5^, in this paper, we report the results of a post-hoc interaction analyses using the same clinical data. Our goal is to highlight through this example when and why subgroup effects are of interest and how they should be conducted, analyzed and interpreted for clinical physicians who are not familiar with statistics. The example we have chosen takes on value in the light of a recent meta-analysis that showed that non-autologous reinforcement of the pancreatic section (except for TachoSil®) after DP reduces the relative risk of POPF^17^.

## Material and methods

Of 631 eligible patients in our original study^5^, enrolled from March 1, 2016, to March 1, 2019, 360 were randomized and 315 patient records were available for analysis (155 in the standard closure group (stapled or hand-sewn pancreatic stump closure, no synthetic or autologous material added to the stump), 160 in the Hemopatch® group (standard closure with Hemopatch® added to the pancreatic stump)). The interaction analysis concerned the 135 patients for whom the pancreatic stump was closed hand-sewn. Once the pancreatic stump was closed (with or without main duct suture closure as per surgeon preference), patients were randomized to affix Hemopatch® to the proximal pancreatic stump or not.

Pancreatic fistula was defined as any measurable volume of drain fluid on or after postoperative day 3, with an amylase content greater than 3 times the upper normal serum value, according to the ISGPS classification^6,7^). Only clinically relevant (Grades B/C postoperative pancreatic fistula (POPF) were considered. Assessment of the onset of POPF was based on clinical, laboratory and/or re-interventional findings during the first 30 post-operative days.

### Ethics approval and consent to participate

As none of patients in the original study were identifiable in this article, no formal consent was requested. However, all patients gave formal consent to participate in the original study. All methods in our original study were carried out in accordance with relevant guidelines and regulations in the Helsinki declaration. The original study was double-blinded: neither the patients nor the independent investigator of outcomes was aware of the allocations. Patients, however, upon request, could be informed of their allocation after the 30-day visit. The Ethikkcommision of the Medical University of Graz approved the study on May 14, 2015 (EC 27–430 ex 14/15). Either local ethic committees, or institutional review boards of all centers approved the study and the study was registered in ClinicalTrials (INS-621000-0760).

### Interaction methodology

The initial step was to determine whether there was interaction between the number and proportion of the dependent event of interest (POPF) among the subgroup population (patients with hand-sewn stump closure and use of Hemopatch®), according to product (T) × (B) = [Hemopatch vs. Basic (T)] × [hand-sewn vs. closure (B)]^4,9–12^.

Next, we calculated the relative excess risk due to interaction (or RERI or the interaction contrast ratio) i.e. the difference between the joint (additive) effect of treatment and a demographic/baseline factor and their effects considered individually^9^. In simple terms, RERI can be calculated as the difference between the (expected) effect based on the summation of the separate effects of the two risk factors under study and the (observed) effect in the joint exposure category. The RERI was calculated according to the formula RERI = RR_T+B+_ − RR_T+B-_ − RR_T-B+_ + 1 where RR_T+B+_ is the relative risk when both risk factors (hand-sewn and Hemopatch®) are present (= 0.47), RR_T+B-_ is the relative risk when one risk factor (T) is present (= 1.24), and RR_T-B+_ is the relative risk when the other risk factor (B) is present (= 1).

Finally, we calculated the “attributable proportion” (AP)^15^ i.e. the proportion of those patients with both exposures (hand-sewn closure and Hemopatch®) who did not sustain a POPF, attributable to the interaction according to the formula AP = RERI/RR_T+B+_^16^.

After the Bonnferroni correction, a p-value of 0.025 or less was considered to be statistically significant.

### Conference presentation

Parts of the contents of this manuscript have been presented at the **IFSES 17th World Congress of Endoscopic Surgery** in Barcelona Nov 27, 2021.

## Results

### Original study

**Figure 1:**
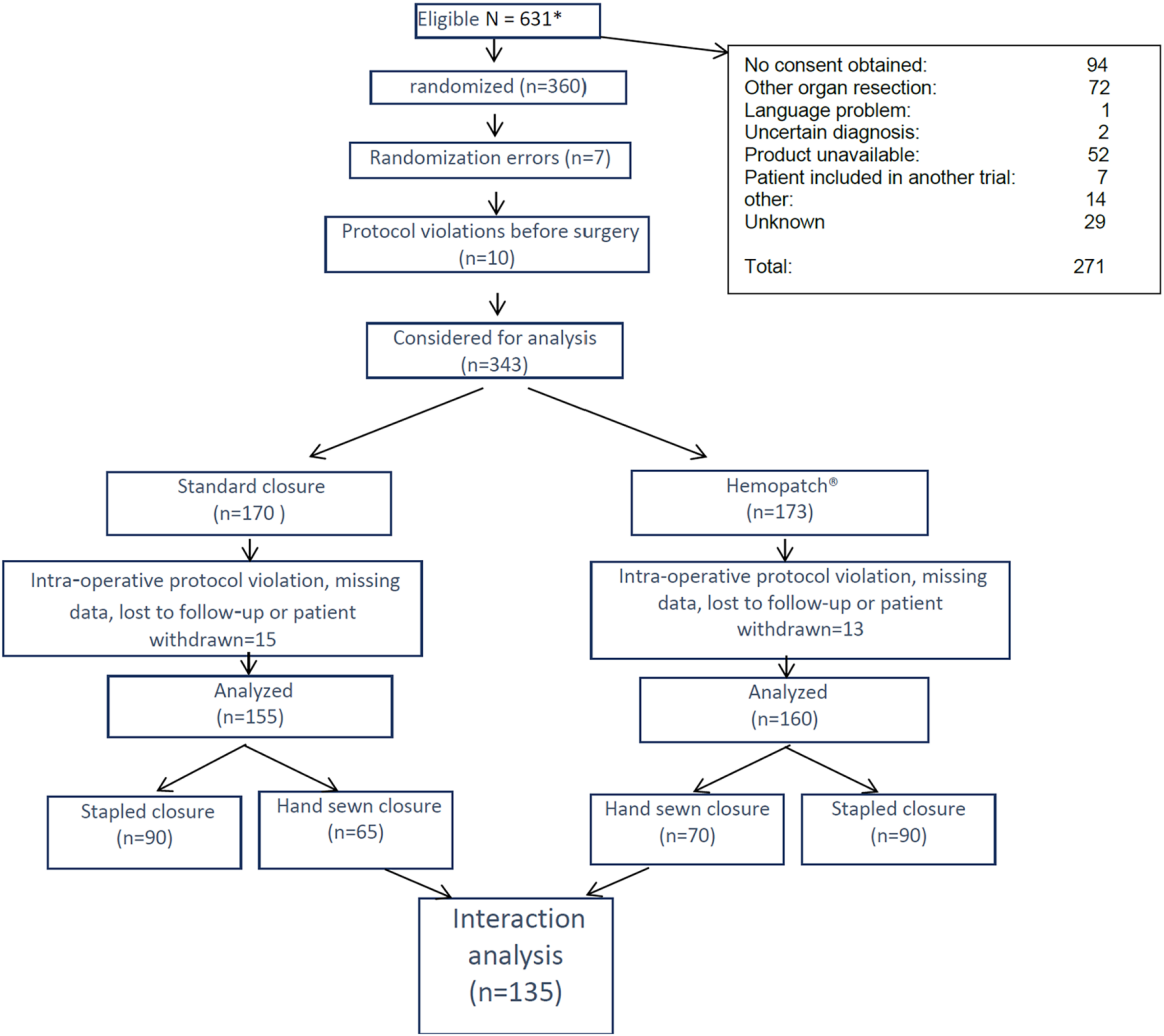
Flow chart of the total population included in the original pancreatic stump trial^5^*. *List of Research sites: Department of Surgery, Medical University of Graz, Graz, Austria; Department of General Surgery, Ruijin Hospital, Shanghai Jiao Tong University School of Medicine, Shanghai Minimally Invasive Surgery Center, Shanghai, P. R. China; Klinik für Allgemein- und Viszeralchirurgie St. Josef-Hospital, Bochum, Germany; Pancreatic Surgery Unit, Humanitas Clinical and Research Center-IRCCS, Rozzano (MI), Italy Humanitas University, Pieve Emanuele (MI), Italy; Division of General and Transplant Surgery, University of Pisa, Pisa, Italy; Department of General and Visceral Surgery, Raphaelsklinik Münster, Münster, Germany; Klinik und Poliklinik für Chirurgie, Klinikum Rechts der Isar, Technische Universität München, München, Germany; Department of General and Pancreatic Surgery—Pancreas Institute, University of Verona, Verona Hospital Trust, Verona, Italy; Department of Visceral, Transplant and Thoracic Surgery, Medical University of Innsbruck, Innsbruck, Austria; Department of Surgery, Salzkammergut-Klinikum,Vöcklabruck, Austria; Clinical Division of General, Visceral and Transplantation Surgery, Medical University of Graz, Austria; Department of Surgery, Campus Bio-Medico University of Rome, Rome, Italy; 2nd Department of Surgery Aretaieio Hospital, National and Kapodistrian University of Athens School of Medicine, Athens, Greece; ##Department of Surgery, Instituto de Investigación Sanitaria Aragón, Miguel Servet University Hospital, Zaragoza, Spain; Department of General Surgery, Faculty of Medicine, Istanbul Medeniyet University Goztepe Training & Research Hospital, Istanbul, Turkey; General Surgery, Fondazione IRCCS Policlinico San Matteo, Pavia, Italy; and Carol Davila University of Medicine and Pharmacy, Bucharest, Romania.

The flow chart of the results of the original study^5^ is provided in Fig. 1.

As there were no statistically significant differences in the rate of POPF according to patient characteristics (sex, age, BMI, ASA score, type of tumor (benign, malignant, neuroendocrine), the number of patients undergoing laparotomy/laparoscopy, duration of operation, number of patients requiring blood transfusion, undergoing chemotherapy, parenchymal texture (normal, friable or intermediate)^5^ or whether the surgeon used absorbable (vs. non-absorbable), interrupted (vs. continuous) sutures, closed the main pancreatic duct or not, or came from a high or low volume center, only two sub-groups from the original study could be considered: (1) hand-sewn pancreatic stump closure (17/65 (26.2%) vs. 7/70 (10.0%)) (Table 1) in patients without or with Hemopatch®, respectively) (*p* = 0.014), (2) closure of the main-pancreatic duct (14/60 (23.3%) vs. 5/65 (7.7%)) without and with the patch, respectively (*p* = 0.015). Only the former was considered for interaction analysis in this study.

**Table 1:** Clinically relevant (B/C) postoperative pancreatic fistula rate according to stump closure technique.

Closure technique	Standard	Standard + Hemopatch®	Total	*p* value
Hand-sewn (n = 135)	17/65 (26.2%)	7/70 (10.0%)	24/135 (17.8%)	0.014
Stapled (n = 180)	19/90 (21.1%)	19/90 (21.1%)	38/180 (21.1%)	0.855*
Total N = 315	36/155 (23.2%)	26/160 (16.3%)	62/315 (19.7%)	0.120

*Yates correction.

### Interaction

While the *p* value of the interaction between the use of Hemopatch® and hand-sewn closure of the pancreatic stump was *p* = 0.034, the RERI of POPF in hand-sewn stump closure − 0.77 (*p* = 0.0204) (Table 2). This means that the probability of having grades B and C POPF in patients who had Hemopatch® added to a hand-sewn stump closure compared to the rate if there were no interaction between Hemopatch® and hand-sewn closure technique was 0.77. The relative risk reduction of grades B and C POPF by the use of Hemoptach® in patients with hand-sewn pancreatic stump closure would be 13%: patients are 13% less likely to sustain a grade B or C POPF because of the interaction between the patch and hand-sewn closure.

**Table 2:** Absolute risks and relative risks of post-operative pancreatic fistula according to strata of technique closure (stapler vs. hand-sewn) and stump reinforcement (Hemopatch® vs. standard (no Hemopatch®)).

	Stapler (B-)		Hand-sewn (B +)		RR
	n	AR	n	AR	
Standard (A−)					
POPF (−)	71		48		
POPF (+)	19	21.1%	17	26.1%	**1.23**
Hemopatch® (A+)					
POPF (−)	71		63		
POPF (+)	19	21.1%	07	10.0%	**0.47**
RR		**1**		**0.383**	

*POPF* post-operative pancreatic fistula, *AR* absolute risk, *RR* relative risk.

Significant values are in [bold].

The AP was − 0.616. This can be interpreted as 61.6% of patients who did not develop POPF did so because of the interaction.

## Discussion

In this study, the unconditional logistic regression analysis (*p* = 0.034) for the interaction term, the additive RERI (= − 0.77) and the attributable proportion (− 0.616) all lead to consider a meaningful protective effect of the addition of Hemopatch® to proximal pancreatic stump hand-sewn closure against the onset of grades B and C POPF. Hemopatch® led to a substantial decrease in POPF compared to when nothing was added to the stump when stump closure was hand-sewn (26.2% vs. 10.0%).

Highlighting the possibilities of using the information contained in subgroup analysis stems from assessing different additive and multiplicative interaction effects, more properly termed “interaction” analysis, rarely performed in the surgical literature.

Interactions may exist between any two or more independent variables in their effects on a dependent variable. In our study, the two independent variables under consideration were hand-sewn stump closure and use of Hemopatch®, while the dependent value was the occurrence of B/C POPF. We concentrated on the effects of the hand-sewn closure only in this paper, as the main duct closure was essentially performed in the hand-sewn closure group which probably explains why both subgroups had a similar tendency to work better in the Hemopatch® group. The fact that the p value of the comparison between laparotomy vs. laparoscopy came out to *p* = 0.075 in the original study^5^ is also compatible with the outcome of the subgroup analysis in hand-sewn closure as most often surgeons use hand-sewn closure in laparotomy rather than laparoscopy^18^. This has its importance as according to a recent survey of 721 surgeons around the world, laparotomy was the preferred route used in distal pancreatectomy for more than three fourths of European and Asian surgeons who responded and just under 60% for surgeons from North and South America^18^. As well, laparotomy was performed in 67% of the European surgeons participating in our recently published study^5^.

The interaction found between hand-sewn pancreatic stump closure and Hemopatch® in the reduction of B/C POPF leads to several hypotheses. Could it be that hand-sewn closure is less traumatic (no crushing effect) on the stump compared to stapled closure, and as such, Hemopatch® is directly in contact with healthy pancreatic parenchyma to exercise its hemostatic effect (as opposed to being in contact with crushed ischemic parenchyma by the jaws of the staple gun), or that Hemopatch® is directly in contact with the parenchyma as opposed to be separated by the staples? Could it be that it is easier to position Hemopatch® over hand-sewn as opposed to stapled closure, especially in open surgery? Could it be that Hemopatch® ensures a mechanical barrier as an intact membrane, as opposed to interruption of the capsule pierced with staples? Could it be that the hemostyptic component of Hemopatch® completes less than optimal hemostasis better after non-stapled, compared to stapled, pancreatic division of the pancreas (found to be more often associated with bleeding than the stapled closure^19^)? The need for intensive or repetitive electrocoagulation for local bleeding might cause parenchymal necrosis, a potential source of fistula could thus be reduced^20^. Indeed, division of the pancreas with cold scalpel or energy-driven devices, including monopolar cautery, is used by many surgeons who do not use staples to divide and close the pancreatic stump. The latter hypothesis is of interest when one considers that there is no formal evidence in favor of fewer B/C POPF between hand-sewn and stapled closure of the pancreatic stump after DP^21^. Another argument might be that because of its sealant property, Hemopatch can seal off microfractures that occur either with sharp division or with the stapling device, especially in “thick” (> 3 cm) pancreases^22^, a factor known to increase the POPF rate^23,24^. Another point of interest is that the PEG-coating on the active side of the patch forms a hydrogel, that covalently binds to proteins on the tissue, ensuring a tight and intimate tissue sealing^20^ and is more resistant towards digestion by activated human pancreatic juice, compared to substances such as fibrin, being a protein rapidly digested by the high protease activity.

According to Sun et al.^1^, the challenges in subgroup analysis concern relative rather than absolute subgroup effects, subgroups that are identifiable at the start of the study, analysis of otherwise methodologically sound studies, and lastly, subgroup effects should not be considered as “all-or-nothing” decisions. Rejecting the all or nothing conclusion, we concur with Sun et al.^1^ in that the “truth” lies somewhere between certainly false and certainly true, and that the effect of Hemopatch® in hand-sewn stump closure is “probably true”, rather than “probably false”.

Two other publications were unable to show any protective effect of Hemopatch® adfixed to the pancreatic stump after distal pancreatectomy^25,26^. However, the results of both of these papers are not comparable to ours. The former^25^ was a single arm, non-comparative, phase 2 (feasibility) study. The second^26^ was a retrospective, historical comparison with a small sample size. The authors included grade A fistula (which we did not), the most frequent of the three types, which might have influenced the outcome. Our study was a randomized clinical trial, with a much higher level of evidence. Three of the authors participated in a meta-analysis comparing different manners to cover the pancreatic stump after DP. The outcomes of nine articles (published between 2009 and 2021), comparing 757 patient who received non-autologous reinforcement and 740 who had no reinforcement were grouped and analyzed. Pooled analysis found a statistically significant lower rate of POPF in the reinforcement group (RR 0.677; 95% CI [0.479, 0.956], *p* = 0.027). The 95% predictive interval ranged from 0.267 to 1.718, showing heterogeneity. This meta-analysis found that non-autologous reinforcement other than with “Tachosil®”, compared to those who did not have any re-enforcement, was effective in reducing the relative risk of POPF, which is in line with the interaction we found in the present manuscript.

Some of the weaknesses of our study have already been mentioned. (1) Our analysis was *post-hoc* with the above-mentioned methodological flaws. However, while the risk of a false positive result due to multiple comparisons is limited (only one additional comparison was used in our study^5^), such comparisons may still have limited ability to guide individual treatment decisions because of multiple variations in population characteristics within the subgroups which are no longer controlled by the original randomization process^10^. (2) The total number of patients being considered is not the number needed for the original power calculation: the subgroup analysis is therefore underpowered and may be fraught with false negative results. (3) Our analysis was *post-hoc* with the above-mentioned methodological flaws: the number of subgroup analyses (K) was determined after the results were known. In this setting, the adequate p value can be determined by the formula 0.05 ÷ K which in our case would be 0.05/2 = 0.025^8^. The *p* value of 0.014 and 0.015 are within this boundary which enforces the idea that there is indeed a statistically interesting relationship between the rate of POPF and the use of Hemopatch® in the population under consideration (hand-sewn stump closure). Moreover, the interaction we found was quantitative and not qualitative (the effect varied only in magnitude, not in direction)^27,28^. This is why we calculated the RERI. (4) Causality has to be interpreted with precaution. The outcome presented here is specific to our study. Although several arguments implying causality can be found in interaction analysis^11^, they are not approved by all. Nonetheless, interaction can be interpreted as causal when (and only if) both the treatment and the baseline factor directly affect the outcome^29,30^, which was the case in our study. However, the causal effect remains speculative because of the above-mentioned weaknesses of an underpowered subgroup, even though our study included a relatively large number of patients (n = 315), and this was a multicenter collaborative effort, reinforcing its generalizability (or external validity). Notwithstanding, true causality can only be proven by a new randomized clinical study with adequate power comparing the effect of Hemopatch® added to the hand-sewn pancreatic stump (vs. no patch) after distal pancreatectomy.

### Step-by-step summary

When subgroup analysis is not pre-specified in randomized studies, results are prone to increased type I error (multiple testing), inadequate power, and can lead to inappropriate statistical interpretation. If subgroups of interest are limited in number, based on strong biological/clinical reasoning or multiple observational data, adjustment for multiple testing is reasonable. Interaction tests should be preferred to subgroup-specific tests; these include: (a) logistic regression to determine the interaction between the number and proportion of the dependent event of interest with the combined independent variables), (b) calculation of the relative excess risk due to interaction, i.e. the difference between the (expected) effect based on the summation of the separate effects of the two risk factors under study and the (observed) effect of the combined exposure and (c) the “attributable proportion” (proportion of patients with the event of interest among those with both exposures that is attributable to their interaction). When all such results concur, the next step is to envisage a specific randomized study where the number of participants and power calculation are based on the subgroup population as a whole.

## Conclusion

The results of our interaction study have their importance in view of the high morbidity associated with POPF after distal pancreatectomy (23% POPF rate)^31^, the unsettled debate concerning the superiority between hand-sewn and stapled stump closure^18,20^ and the predominant use of laparotomy world-wide for distal pancreatectomy, where hand-sewn closure is performed in 15%, 32%, 45%, and 31% of cases for surgeons in North America/Canada, South/Central America, Europe/Africa and Asia/Oceania, respectively^18^. It is important that trialists, reviewers, and editors understand these issues^10^ to correctly distinguish between primary (i.e. hypothesis testing) and secondary (i.e. hypothesis generating) subgroup analyses^29,30^. The former, when positive, can directly affect patient care; the latter only requires confirmation*.* Finally, the best way to validate a statistical interaction is to replicate the comparison in subsequent specific, properly powered randomized trials composed of only the subgroup population where the interaction was found^9^. In the future, a stratified randomized study of only hand-sewn pancreatic stump closed distal pancreatectomies might provide more methodologically sound data.

## Data Availability

All data in this study are available upon demand from A Fingerhut. The datasets generated and/or analyzed during the current study are not publicly available because the monitoring company that detained the data sets (Medpass) disappeared during the COVID 19 pandemic. Partial data sets are available from the corresponding author on reasonable request.
